# A Thymidine Kinase recombinant protein-based ELISA for detecting antibodies to Duck Plague Virus

**DOI:** 10.1186/1743-422X-7-77

**Published:** 2010-04-23

**Authors:** Yongping Wen, Anchun Cheng, Mingshu Wang, Han Ge, Chanjuan Shen, Sitong Liu, Jun Xiang, Renyong Jia, Dekang Zhu, Xiaoyue Chen, Bei Lian, Hua Chang, Yi Zhou

**Affiliations:** 1 Avian Diseases Research Center, College of Veterinary Medicine of Sichuan Agricultural University, Ya'an, Sichuan, 625014, China; 2 Key Laboratory of Animal Diseases and Human Health of Sichuan Province, Ya'an, Sichuan, 625014, China; 3 Epizootic Diseases Institute of Sichuan Agricultural University, Ya'an, Sichuan, 625014, China

## Abstract

**Background:**

Duck plague virus (DPV) is the causative agent of Duck Plague (DP) that causes significant morbidity and mortality throughout duck-producing areas of the world. The diagnosis of DP currently relies on the use of live or inactivated whole DPV virion as antigens in ELISA, but it is too laborious and expensive for routine application, and it is still difficult to get purified DPV virion with current technology.

**Results:**

In this study, we describe the expression and purification of a recombinant Thymidine Kinase (TK) protein which makes antigen in an in-house developed, optimized and standardized ELISA. The specificity of the optimized TK-ELISA was evaluated by antisera against Duck Plague Virus (DPV), Duck Hepatitis B Virus (DHBV), Duck Hepatitis Virus (DHV), *Riemerella Anatipestifer*(*R. A*), *Escherichia coli *(*E. coli*) and *Salmonella anatum *(*S. anatum*). Only antisera against DPV yielded a specific and strong signal. In order to determine the sensitivity of the TK-ELISA, a panel of diluted sera was tested, and the minimum detection limit of 1:2560 (OD450 nm = 0.401) was obtained according to the endpoint cut-off (0.2438). The repeatability and reproducibility under the experimental conditions demonstrates a low variability (P > 0.05). The suspected sera samples (n = 30) were determined by TK-ELISA and the positive rate is 90% (27/30), and the TK-ELISA showed 83.33% (22+3/30) coincidence rate with the Serum Neutralization Test (SNT) and 90% (24+3/30) coincidence rate with the whole DPV virion based-ELISA (DPV-ELISA). When defining the dynamics of antibody response to attenuated live DPV vaccine, the maximum antibodies is reached after 4 weeks.

**Conclusions:**

The results suggest that the TK-ELISA provides high specificity, sensitivity, repeatability and reproducibility for detection of anti-DPV antibodies in duck sera, and has the potential to be much simpler than DPV-ELISA and SNT for the sera epidemiological investigation.

## Background

Duck plague (DP), which is caused by Duck Plague Virus (DPV), is an acute, contagious and lethal disease discovered, firstly, in Holland [1]. DPV is currently classified belonging to the Alphaherpesvirinae subfamily of the herpesvirus family but has not been grouped into any genus yet [2]. There are 34 species within Order Anseriformes' host range. Ducks, geese, and swans are the susceptive species to DP [3]. The characteristics of DP are vascular damage, tissue hemorrhages, digestive mucosal eruptions, lesions of lymphoid organs, and degenerative changes in parenchymatous organ [4,5]. Considerable economic losses were suffered by the DP in duck-producing areas of the world [6,7]. China, holding the largest population of waterfowl [8], was also inflicted with heavy losses attributing to the outbreak of DP, since it was firstly reported by Huang [9]. Therefore, to develop a fast and available diagnose method to predict the infection of DPV in a suspected flock, on farm, is extremely urgent.

The diagnosis of DP may be made on the basis of characteristic internal lesions and final diagnosis can be made by virus isolation and identification [10,11], however, it is laborious and time-consuming. In recent years, the Fluorescent Quantitative Real-Time PCR (FQ-PCR) [12], Loop-Mediated Isothermal Amplification(LAMP) [13], Antigen-Capture Enzyme-Linked Immunosorbent Assay (AC-ELISA) [14], Histopathology [15] and Electron Microscopy [16] have been developed quickly. Whereas the key of prevention and control is more than clinical diagnosis, the vaccination, also a critical factor, is generally considered to be the most effective and financially viable method of preventing infectious diseases. In vaccination against DP, antibody detection plays an extremely important role. It is used to detect (subclinical) field infections, to check the response to vaccines and predict the optimal age for vaccination. The gold standard assay for DP antibody detection is the Neutralization Test (NT) [17]. However, the NT requires maintenance and use of live virus and cell cultures and must be performed under aseptic conditions, furthermore, it requires up to 3 days for results. Until now the ELISA assays have been developed for antibody or antigen detection [18-20] and the whole DPV virion usually acts as antigen for the detection of antibodies against DPV in the indirect ELISA assay (I-ELISA) [21]. But much time and energy must be paid in getting the virion as the coating antigen. Compared with the DPV-ELISA, the development of the TK-ELISA assay in this paper is more economical and more convenient.

The DPV gene library has been constructed and identified [22]. The Thymidine Kinase (TK) gene of DPV has been successfully cloned [23]. Comparison with other available Herpesvirus, TK sequences reveals a high homology to those of the Alphaherpesviruses [23-26]. The research also shows that the TK gene, considered the main virulence gene [27,28], is dispensable during the process of virus multiplication [29]. Therefore, the aim of this paper was to describe the expression of a recombinant TK protein, which is applied in antigen coating, in an in-house developed, optimized and standardized ELISA. To date, there are no reports of using expressed DPV recombinant protein acting the same role. To the best of our knowledge, this is the first report of expressing TK protein of DPV in *Escherichia coli *(*E. coli*) and the development of an indirect ELISA test.

## Results

### Expression of the recombinant DPV TK protein

**Figure 1:**
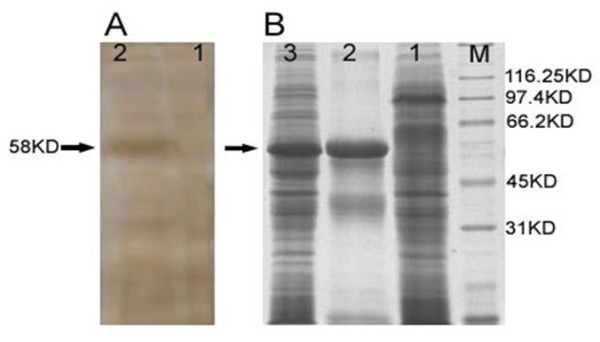
**^B^The SDS-PAGE analysis of recombinant TK produced in *E. coil***. M: Protein Marker; Lane 1. uninduced; Lane 2. The purified protein; Lane 3. The unpurified protein. ^A^**Identifiction of the TK protein by Western Blot**. Lane 1: uninduced; lane 2: IPTG induced. The arrows indicate the fusion protein of pET-TK.

The product was expressed in *E. coli *system as a His6-tagged recombinant TK fusion protein of approximately 58 KD (Fig. 1.B, lane 2,3). Western Blot analysis showed that the purified His6-tagged TK was recognized by the rabbit anti-DPV IgG with a specific signal at 58 KD; which is the expected size of the fusion protein (Fig. 1.A, lane 2). However, when using the negative control serum, no positive signal was observed (Fig. 1.A, lane 1); indicating that the recombinant protein induced an immunological response and thus the antiserum had a high level of specificity.

### Optimization of ELISA Procedure

**Figure 2:**
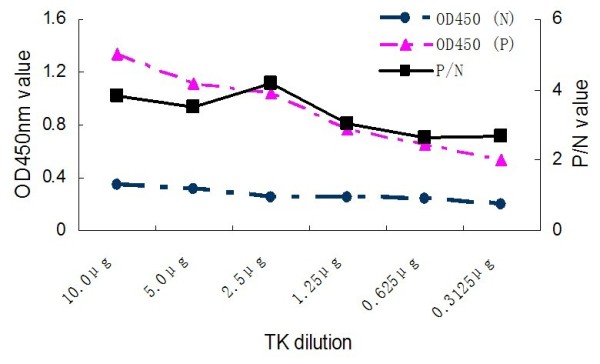
**Serial dilution of antigens**.

**Figure 3:**
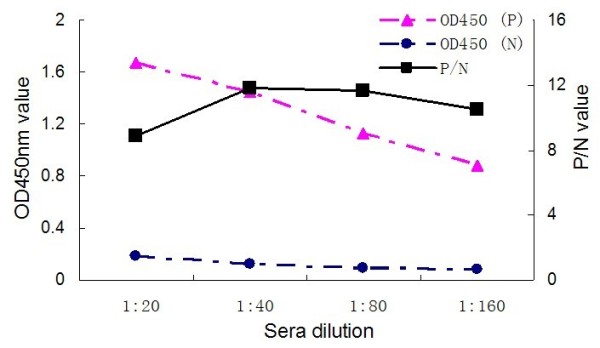
**Anti-duck horseradish peroxidase secondary antibody diluted to 1: 1000**.

**Figure 4:**
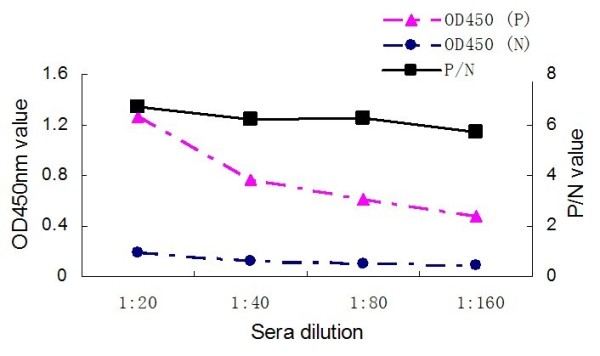
**Anti-duck horseradish peroxidase secondary antibody diluted to 1: 2000**.

**Figure 5:**
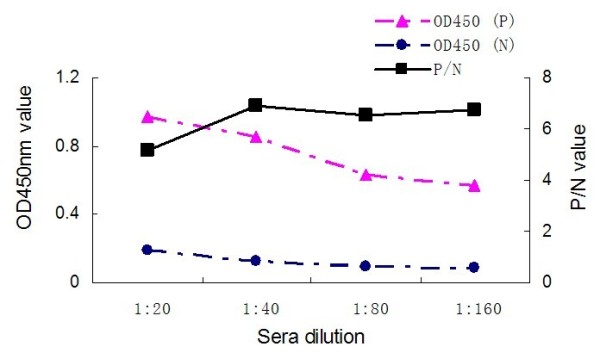
**Anti-duck horseradish peroxidase secondary antibody diluted to 1: 4000**.

The coating antigen and serum dilution corresponding to the highest P/N value (ODtest sample/ODnegative control) were regarded as optimal antigen concentration and optimal serum dilution. The optimal antigen concentration of TK protein, anti-duck horseradish peroxidise secondary antibody dilution and the serum dilution were found by checkerboard titration at 2.5 μg/100 μl (1:200) (Fig. 2), 1:2000 and 1:80 (Fig. 4), respectively. The process of optimization of the anti-duck horseradish peroxidase secondary antibody and sera dilutions was revealed in the Fig. 3, 4 and 5.

### The endpoint cut-off value

The results of intra-day and inter-day assays analyzed by SAS8.22 showed the difference among 6 groups was not significant (P > 0.05). The OD_450 nm_average values of 0.1211 was acquired after these duck-negative sera samples (n = 30) were detected by the TK-ELISA with a standard deviation of 0.0409. The endpoint cut-off value of the ELISA was calculated according to a Gaussian population distribution [30]. Consequently, for a 99% confidence interval, the endpoint cut-off value was defined as follows: mean of the negative serum OD_450 nm _values plus three standard deviations = 0.1211 + 3 × 0.0409 = 0.2438.

### Analytical specificity and sensitivity of the TK-ELISA

**Figure 6:**
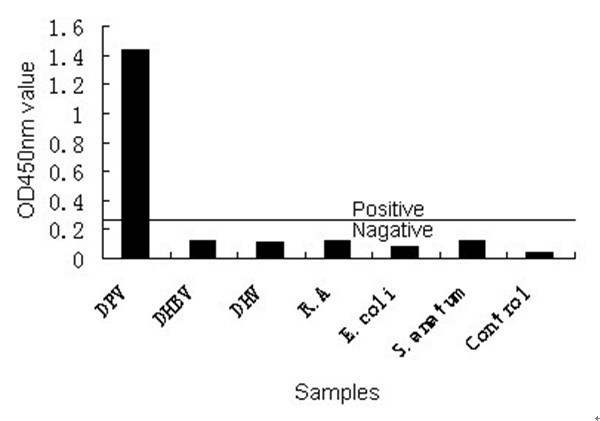
**Analytical specificity of the TK-ELISA**.

On the basis of endpoint cut-off value (0.2438), the OD_450 nm _value of the DPV-positive sera is 1.398 and 1.432 both of which were higher than 0.2438, therefore, positive. The OD450 nm value of the other samples were all lower than 0.2438. The results indicated that TK-ELISA had a high specificity for the DPV-positive sera and there was no cross-reaction with antisera against Duck Hepatitis B Virus (DHBV), Duck Hepatitis Virus (DHV), *Riemerella Anatipestifer *(*R. A*), *Escherichia coli *(*E. coli*) and *Salmonella anatum *(*S. anatum*) (Fig. 6).

After evaluating the sensitivity of a panel of diluted sera, a minimum detection limit of 1:2560 (OD450 nm = 0.401) was obtained according to the endpoint cut-off (0.2438), but the blank control did not yield positive results (Table 1).

**Table 1:** One positive serum sample with gradient dilution to evaluate the sensitivity of the TK-ELISA.

Dilution	1:160	1:320	1:640	1:1280	1:2560	1:5120	1:10240
**OD450 nm**	1.432	0.984	0.975	0.542	0.401	0.107	0.111

**P or N**	+	+	+	+	+	-	-

The sera-positive and sera-negative are marked with a plus (+) or a minus (-).

### Repeatability and reproducibility of the TK-ELISA

The results analyzed by SAS8.22 showed the difference among 3 groups was not significant (P > 0.05). Table 2, 3 shows the repeatability and reproducibility of the assay under the experimental conditions, demonstrating the low variability.

**Table 2:** The repeatability of the assay.

**NO**.	Number of replications						X	SD	C.V(%)
				
	1	2	3	4	5	6			
**A**	1.322	1.485	1.409	1.388	1.425	1.605	1.439	0.097	6.742
**B**	1.611	1.569	1.398	1.487	1.552	1.623	1.540	0.085	5.501
**C**	1.324	1.382	1.578	1.418	1.308	1.509	1.419	0.106	7.459

**Table 3:** The reproducibility of the assay.

**Days**.	**NO**.			X	SD	C.V(%)
				
	A	B	C			
**1**	1.401	1.609	1.535	1.515	0.105	6.959
**2**	1.602	1.498	1.487	1.529	0.063	4.150
**3**	1.384	1.332	1.517	1.411	0.095	6.762

### Analytical inhibition of the TK-ELISA

**Figure 7:**
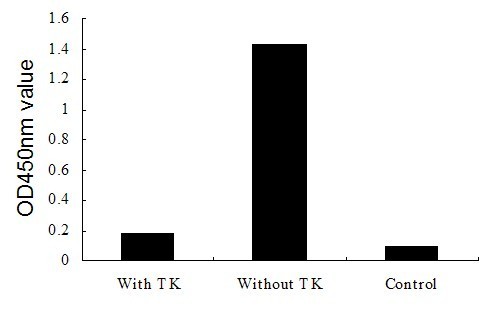
**Analytical inhibition of the TK-ELISA**.

After repeating three times using the TK-ELISA, it was observed that the OD_450 nm _values in these reactions declined sharply by more than 50% after blocking with TK protein (Fig. 7). This result indicates that the assay was highly specific for DPV-TK antigen.

### The coincidence rate among SNT, TK-ELISA and DPV-ELISA

By the TK-ELISA, the SNT and DPV-ELISA, the suspected sera samples (n = 30) were determined, respectively. The results showed that the positive rate were 90% (27/30), 80% (24/30) and 73.3% (22/30), respectively. In addition, the TK-ELISA showed 83.33% (22+3/30) coincidence rate with the SNT and 90% (24+3/30) coincidence rate with the DPV-ELISA (Table 4).

**Table 4:** The coincidence rate among SNT, TK-ELISA and DPV-ELISA for detection of antibodies to DPV.

	SNT		DPV-ELISA		
**TK-ELISA**	**Positive**	**Negative**	**Positive**	**Negative**	**Total**

Positive	22	5	24	3	27
Negative	0	3	0	3	3
Total	22	8	24	6	30

The coincidence rate was calculated by the formula: (no. of samples positive by both method + no. of samples negative by both methods)/total no. samples× 100.

### The kinetics of antibody production

**Figure 8:**
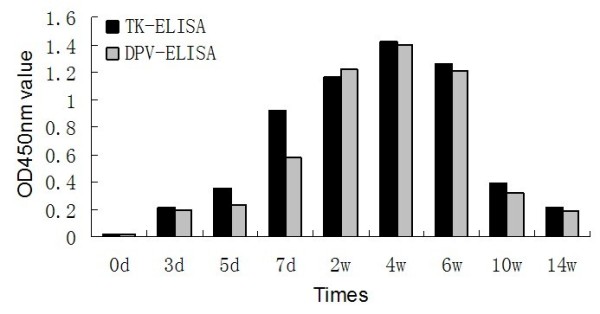
**The kinetics of antibody production using TK-ELISA and DPV-ELISA**.

The dynamics of antibody response to attenuated live DPV vaccine for duck using the TK-ELISA and the DPV-ELISA are shown in Fig 8. In terms of TK-ELISA, the OD_450 nm _values started to rise at approximately day 5, and after inoculation reaching maximum value at week 4, followed, after, by their gradual decrease. But as for DPV-ELISA, the OD_450 nm _values started to rise at approximately day 7, which is a bit later than that of TK-ELISA.

## Discussion

In this study, the TK recombinant protein was produced in *E. coli *system to replace the whole DPV virion used for detecting antibodies in TK-ELISA. The use of un-purified whole virion preparation in the I-ELISA, brings high background absorbance but purification of the virion is laborious and expensive [31]. Therefore, many people choose the recombinant protein which has advantages over whole virion as antigen for the detection of antibodies [32-35]. Firstly, the recombinant protein antigen is not infectious, so it gives the opportunity to guarantee DPV serodiagnostic assays rapidly and safely without virus containment facilities. Secondly, the recombinant protein antigen could be easily produced in general laboratory without any limitation. Particularly, with the help of recombinant antigen coated on the plates, specificity of the ELISA was greatly improved. This enhanced specificity is especially important for eradication programs in developing countries where true-positive results are often interfered due to the deficient conditions of sera transport and storage; one of the most common reasons for unspecific results in ELISA [36].

The TK gene, which is the main virulence gene and dispensable gene for herpesvirus multiplication, is essential for latent infection [37,38]. Consequently, the TK gene is often considered to be the prior target gene for constructing the gene-deleted vaccine. Based on the deletion of TK gene, the vaccine of Pseudorabies Virus (PRV) [39] and Bovine Herpesvirus (BHV) [40] have been developed successfully. It suggests that preventing and controlling DP may depend on the TK gene-deleted vaccine in future. Previously, the related reports present that the protein-based ELISA can be used to distinguish antibodies induced by the wild-type virus strain infection from those induced by vaccined animals [41-43]. Therefore, if DPV TK gene-deleted vaccine was developed successfully, the TK-ELISA discussed above would significantly contribute to the diagnosis methods of discrimination between the infection of DPV TK gene-deleted vaccine strain and that of wild-type DPV strain. Similar to the PRV eradication programs, it can strongly support that of DPV in future.

Another, the *E. coli *system, was chosen to produce the TK protein. The many advantages of *E. coli *have ensured that it remains a valuable organism for the high-level production of recombinant proteins [44,45]. The TK protein, which is highly conserved among DPV can be used as candidate diagnostic antigen because of its group and type specificity. Purification of expressed TK protein by Nickel Affinity Chromatography is able to insure the unique component of coating protein, as well as the decrease of non-specific binding. Furthermore, the purified protein was gradient dialysed to reduce concentration of urea which is added at the time of dissolving inclusion bodies [46]. Thus, the renaturation of recombinant protein makes good antigenicity for coating antigen. To enhance the specificity and sensitivity of the TK-ELISA, the dilution of the TK protein and anti-duck horseradish peroxidase secondary antibody were screened and the optimal dilutions are 2.5 μg/100 μl (1:200) and 1:2 000, respectively. What's more, Steinitz et al. [47] presents a practical approach to defining the conditions on optimal coating, such as coating buffer, temperature and duration of coating. However, although there is much research into the effects of temperature and duration of coating, there is little research into the effects of coating buffer. In this study, in order to get the optimal coating buffer, 0.05 M bicarbonate/carbonate buffer (pH 9.6), Phosphate Buffered Saline (pH 7.4 and 8.5) were screened. It was discovered that 0.05 M bicarbonate/carbonate buffer (pH 9.6) was the best coating buffer for immobilization of the protein. Otherwise, we have found that coating plates at 4°C overnight also reduced the background.

Interestingly as an additional discovery, the DPV-specific antibodies could be clearly detected approximately 5 days earlier than the conventional assay after attenuated live DPV vaccine-induced immunity. Most likely, TK gene is a part of early genes of herpesvirus, in which TK genes share the high conservation [48,49]. The TK, as the key enzyme in replication of DPV, is probably expressed at early times after infection, which caused its rapid immune recognition after the infection.

In summary; in this study, the TK-ELISA manages to provide an new approach to screening and studying DPV, and this method could replace DPV-ELISA and SNT as an alternative, sometimes prove even more effective.

## Conclusions

This paper demonstrated the expression of the TK protein of DPV in *E. coli *system and their application in developing an indirect TK-ELISA to detect anti-DPV antibodies from DPV-infected duck sera. The TK-ELISA, as a simple and economical method, may exert a strong influence on DPV eradication campaigns and control programs in duck-producing areas of the world.

## Methods

### Sera samples

A panel of sera containing 70 duck sera samples, of which 5 were DPV-positive, 35 were DPV-negative, and remaining 30 were suspected infectious DPV, was used in this study. The 30 suspected DPV-positive sera samples were obtained from clinical samples. The 5 DPV-positive sera were acquired by inoculation of DPV Virulence Strain. 30 of the sera samples from ducks uninfected with DPV (DPV-negative) were used to evaluate the endpoint cut-off value of TK-ELISA, and the remaining 5 DPV-negative serum were used to optimize the procedure of TK-ELISA. To calculate the specificity of the TK-ELISA 20 serum samples tested positive for DHBV, DHV, *R. A, E. coli*, and infectious *S. anatum *were also used. To study the kinetics of antibody production 20 serum samples were obtained by inoculating a new different group of DPV infected ducks subcutaneously with the attenuated live DPV vaccine. All the test sera above were collected and were preserved at -70°C in author's laboratory until used.

### Expression and purification of recombinant DPV TK protein

The pET-32a(+)/Tk expression plasmid was provided by the author's laboratory [23]. The pET-32a(+)-Tk was transformed into *E. coli *BL21(DE3) and the recombinant clones were selected on Luria bertani (LB) agar plates containing ampicillin (100 mg/ml). Transformants grew on LB plates containing ampicillin (100 μg/ml) at 37°C for 18 h. A single colony from the culture was inoculated into LB medium with ampicillin. When the cultures initially reached logarithmic phase (at OD600 of 0.5-0.6), protein expression was induced at 37°C by isopropyl-β-D-thiogalactoside (IPTG) (final concentration 0.6 mM) for a further 4 hours. The recombinant His-tagged protein were purified by nickel affinity chromatography according to the manufacturer's protocol (Bio-Rad) and the samples were stored at -70°C until use.

### The Western Blot assay

To identify the expression of TK protein, the SDS-PAGE and Western Blotting assay were carried out according to the standard procedure. Antibodies against the DPV were raised in rabbits [50]. Briefly, the protein samples were electrophoresed in SDS-PAGE (12%) and subsequently transferred to PVDF membrane [51]. Then, Membrane was incubated with rabbit anti-DPV antibodies (1:200 dilution) overnight at 4°C. After washing three times with PBS-T (0.2% Tween 20 in PBS, PH 7.4), the second incubation of membrane was accomplished with horseradish peroxidase-linked goat anti-rabbit immunoglobulin G (IgG) (Amersham) for 1 hour at 37°C. As a result, the specific band was detected using DAB Enhancer Reagent Solution (Beijing Zhong Shan-Golden Bridge Biological Technology Co., Ltd., China).

### Procedures for the TK-ELISA

The DPV-positive by inoculation of DPV Virulence Strain and the DPV-negative duck sera obtained from our laboratory were used to optimize the TK-ELISA. The 96-Well Microtiter Plates (Nunc, Denmark) were coated with 100 μl dilution of purified TK protein in sodium bicarbonate buffer (pH 9.6), and were incubated at 4°C overnight. The optimal antigen concentration and the optimal serum dilution were determined by checkerboard titration of a coating antigen sample with gradient dilution (1:50, 1:100, 1:200, 1:400, 1:800, 1:1600) and an antisera sample with gradient dilution (1:20, 1:40, 1:80, 1:160). Plates were washed three times with PBS containing 0.05% Tween-20 (PBST) and then incubated with 100 μl of blocking solution (1% BSA in PBST) for 60 min at 37°C. Before adding the 100 μl of positive and negative diluted sera respectively, the plates, again, were washed and then incubated for 60 min at 37°C. After washing with PBST, 100 μl PBS diluted (1:1000, 1:2000, 1:4000), anti-duck horseradish peroxidase (KPL, Gaithersburg, USA) secondary antibody was added and the plates were incubated at 37°C for 45 min. Following the incubation, the plates were again washed as described above, and 100 μl/well of 3,3', 5,5'-etramethylbenzidine (TMB) was added. After 20 minutes incubation at 37°C, the reaction was stopped by addition of 50 μl of 2 mol/l H_2_SO_4_. The optical density (OD) was measured at 450 nm, using a Bio-Rad Model 860 Plate Reader (Bio-Rad, CA, USA).

### The endpoint cut-off value

The 30 duck-negative sera samples were used to establish the endpoint cut-off value. The ELISA plates were coated with optimal TK antigen concentration and these sera samples at the optimal dilution were added. The TK-ELISA was performed in the optimal procedure described above. At the same time, the 0.01 M PBS was used for the blank control. Intra-day and inter-day variability were estimated by assaying sera samples containing 30 different duck-negative sera samples 6 times on the same day and on 3 separate days [52]. The data of result was analyzed with General Linear Model (GLM) procedures of SAS8.22, and the value was presented as least squares means± standard deviation. The significant differences of least square means were tested with the Duncan Test (*P *< 0.01). The cut-off value was calculated using the formula: mean of the negative serum values plus three standard deviations (SDs) [32].

### Analytical specificity and sensitivity of the TK-ELISA

As stated above; the specificity of TK-ELISA was evaluated by testing with a panel of sera containing antibodies against DHBV, DHV, *R. A, E. coli*, and *S. anatum*. Other groups; the DPV-positive sera and DPV-negative sera were used as control groups respectively.

The 96-Well Microtiter Plates (Nunc, Denmark) were coated with 100 μl of a 1:200 (25 μg/100 μl) dilution of purified TK protein in sodium bicarbonate buffer (pH 9.6), and the seven diluted sera (1:160, 1:320, 1:640, 1:1280, 1:2560, 1:5120, 1:10,240) were added. The other operating conditions were carried out according to the optimal procedure described above. Therefore, the sensitivity of the TK-ELISA was inferred.

### Analytical repeatability and reproducibility of TK-ELISA

Based on the validation of Serological Assays, the coefficient of variation (C.V.) for six replicates of 3 specimens were calculated for repeatability, these, according to TK-ELISA procedure were run on the same day. Also, the reproducibility of the set of specimens was analyzed in 3 runs during 3 days [53]. The data of result was analyzed with General Linear Model (GLM) procedures of SAS8.22, and the value was presented as least squares means± standard deviation. The significant differences of least square means were tested with the Duncan Test.

### Analytical inhibition of the TK-ELISA

Firstly, the DPV positive serum and the TK protein were diluted, respectively, with PBS, according to the optimal dilution (described above). Secondly, the dilution of serum was divided into two groups; A and B. Thirdly, the 50 μl dilution of TK protein was mixed with an equal volume of A (50 μl), and then the mixture was incubated for 60 min at 37°C. At last, the mixtures were tested in the TK-ELISA by adding 100 μl of each mixture, and the assay was performed according to the description above. At the same time, a 100 μl B was performed as the negative control and was also tested. The OD value was converted to percentage inhibition (PI) value using the formula [32]: PI(%) = 100× [1-(ODtest serum/ODcontrol)], where ODcontrol is the mean OD of wells containing positive serum alone.

### The coincidence rate among SNT, TK-ELISA and DPV-ELISA

The suspected sera samples (n = 30) for infectious DPV were collected and preserved in our laboratory. These sera samples were tested by the TK-ELISA which is discussed above, SNT and the DPV-ELISA, respectively. The SNT was carried out for these samples in a Six-Well Cell Culture Plates with duck embryo fibroblast cells. Duplicates of serial twofold dilutions of sera, inactivated at 56°C for 30 min were tested as previously described [54]. Titres were expressed as the reciprocal of the serum dilution that inhibited 75% of viral cytopathic effect. A serum sample was considered positive when it had a titer of = log101.0, equivalent to a serum dilution = 1:10. The DPV-ELISA was carried out for these samples referring to Xuefeng et al. [21]. Therefore, the coincidence rates were calculated from them.

### Evaluation for the kinetics of antibody production using TK-ELISA and DPV-ELISA

Sera (n = 20) from twenty duck subcutaneously inoculated with attenuated live DPV vaccine (offered by author's laboratory), taken on days 0, 3, 5, 7 and weeks 2, 4, 6, 10, 14 after inoculation, were used by TK-ELISA and DPV-ELISA to study the kinetics of antibody production.

## Competing interests

The authors declare that they have no competing interests.

## Authors' contributions

YW carried out most of the experiments and wrote the manuscript. AC and MW critically revised the experiment design and the manuscript. HG, CS, SL, JX, RJ, DZ, XC, BL, HC and YZ helped with the experiment. All the authors read and approved the final manuscript.
